# Supplementary material for: Association of cancer with overactive bladder and impact of overactive bladder on mortality among cancer survivors: NHANES 1999-2018
Source: PLoS One. 2025 Apr 15;20(4):e0320491. doi: 10.1371/journal.pone.0320491 (PMC11999114; doi:10.1371/journal.pone.0320491)
Supplement: Table S1 — (DOCX) [file pone.0320491.s001.docx]

**Table** **S1.** Relationship between Non-GU and GU cancer and overactive bladder among participants.

| **Variable** | **OR (95% CI)** | ***P* value** |
| --- | --- | --- |
| Cancer |  |  |
| No | ref | ref |
| Non-GU | 1.37 (1.21, 1.56) | < 0.0001 |
| GU | 2.04 (1.61, 2.58) | < 0.0001 |
| Sex |  |  |
| Female | ref | ref |
| Male | 0.58 (0.52, 0.64) | < 0.0001 |
| Age group |  |  |
| ≤49 | ref | ref |
| 50-65 | 2.34 (2.08, 2.63) | < 0.0001 |
| ≥65 | 4.18 (3.65, 4.78) | < 0.0001 |
| Race |  |  |
| Hispanic | ref | ref |
| Non-Hispanic White | 0.82 (0.71, 0.95) | 0.01 |
| Non-Hispanic Black | 1.52 (1.31, 1.77) | < 0.0001 |
| Mexican American | 0.87 (0.74, 1.01) | 0.07 |
| Other | 0.85 (0.69, 1.04) | 0.11 |
| Education |  |  |
| Less than high school | ref | ref |
| High school or equivalent | 0.67 (0.60, 0.74) | < 0.0001 |
| Some college or AA degree | 0.62 (0.55, 0.70) | < 0.0001 |
| College graduate or above | 0.47 (0.40, 0.54) | < 0.0001 |
| Marital status |  |  |
| Divorced | ref | ref |
| Living with partner | 0.95 (0.80, 1.13) | 0.58 |
| Married | 0.84 (0.75, 0.95) | 0.005 |
| Never married | 0.91 (0.78, 1.05) | 0.19 |
| Separated | 1.28 (1.04, 1.56) | 0.02 |
| Widowed | 0.92 (0.77, 1.11) | 0.40 |
| BMI category |  |  |
| <25 | ref | ref |
| 25-30 | 1.20 (1.08, 1.33) | < 0.001 |
| ≥30 | 1.66 (1.50, 1.84) | < 0.0001 |
| Smoking status |  |  |
| Never | ref | ref |
| Former | 1.14 (0.99, 1.31) | 0.08 |
| Now | 1.44 (1.28, 1.63) | < 0.0001 |
| Drinking status |  |  |
| Never | ref | ref |
| Former | 1.12 (0.96, 1.30) | 0.16 |
| Now | 0.86 (0.74, 0.99) | 0.03 |
| Hypertension |  |  |
| No | ref | ref |
| Yes | 1.35 (1.23, 1.49) | < 0.0001 |
| Diabetes |  |  |
| No | ref | ref |
| IGT | 1.06 (0.88, 1.27) | 0.54 |
| IFG | 1.28 (1.02, 1.61) | 0.03 |
| DM | 1.51 (1.36, 1.68) | < 0.0001 |

BMI, body mass index; CI, confidence interval; DM, diabetes mellitus; IFG, impaired fasting glycaemia; IGT, impaired glucose tolerance; OR, odds ratio.

Model adjusted for demographic characteristics (sex, age group, race, education, marital status); BMI category, smoking status, drinking status, hypertension and diabetes.

GU cancer: genitourinary cancer, including cancers of the kidney, prostate, bladder, and testis.

Non-GU cancer: non-genitourinary cancer, including other kinds of cancer in addition to GU cancer.
