# Supplementary material for: Association of cancer with overactive bladder and impact of overactive bladder on mortality among cancer survivors: NHANES 1999-2018
Source: PLoS One. 2025 Apr 15;20(4):e0320491. doi: 10.1371/journal.pone.0320491 (PMC11999114; doi:10.1371/journal.pone.0320491)
Supplement: Table S2 — (DOCX) [file pone.0320491.s002.docx]

**Table S2.** Relationship between non-pelvic and pelvic cancer and overactive bladder among participants.

| **Variable** | **OR (95% CI)** | ***P* value** |
| --- | --- | --- |
| Cancer |  |  |
| No | ref | ref |
| Non-pelvic | 1.30 (1.12, 1.51) | < 0.001 |
| Pelvic | 1.84 (1.54, 2.20) | < 0.0001 |
| Sex |  |  |
| Female | ref | ref |
| Male | 0.59 (0.53, 0.64) | < 0.0001 |
| Age group |  |  |
| ≤49 | ref | ref |
| 50-65 | 2.35 (2.09, 2.64) | < 0.0001 |
| ≥65 | 4.24 (3.71, 4.84) | < 0.0001 |
| Race |  |  |
| Hispanic | ref | ref |
| Non-Hispanic White | 0.82 (0.71, 0.96) | 0.01 |
| Non-Hispanic Black | 1.53 (1.32, 1.78) | < 0.0001 |
| Mexican American | 0.87 (0.74, 1.02) | 0.08 |
| Other | 0.85 (0.69, 1.03) | 0.10 |
| Education |  |  |
| Less than high school | ref | ref |
| High school or equivalent | 0.67 (0.60, 0.74) | < 0.0001 |
| Some college or AA degree | 0.62 (0.55, 0.70) | < 0.0001 |
| College graduate or above | 0.47 (0.41, 0.54) | < 0.0001 |
| Marital status |  |  |
| Divorced | ref | ref |
| Living with partner | 0.96 (0.80, 1.14) | 0.61 |
| Married | 0.85 (0.75, 0.95) | 0.005 |
| Never married | 0.91 (0.79, 1.05) | 0.20 |
| Separated | 1.28 (1.04, 1.56) | 0.02 |
| Widowed | 0.92 (0.77, 1.11) | 0.38 |
| BMI category |  |  |
| <25 | ref | ref |
| 25-30 | 1.20 (1.08, 1.33) | < 0.001 |
| ≥30 | 1.66 (1.50, 1.84) | < 0.0001 |
| Smoking status |  |  |
| Never | ref | ref |
| Former | 1.14 (0.98, 1.31) | 0.08 |
| Now | 1.43 (1.27, 1.62) | < 0.0001 |
| Drinking status |  |  |
| Never | ref | ref |
| Former | 1.11 (0.96, 1.30) | 0.16 |
| Now | 0.86 (0.74, 0.99) | 0.03 |
| Hypertension |  |  |
| No | ref | ref |
| Yes | 1.35 (1.23, 1.49) | < 0.0001 |
| Diabetes |  |  |
| No | ref | ref |
| IGT | 1.06 (0.88, 1.27) | 0.53 |
| IFG | 1.28 (1.02, 1.61) | 0.03 |
| DM | 1.51 (1.36, 1.68) | < 0.0001 |

BMI, body mass index; CI, confidence interval; DM, diabetes mellitus; IFG, impaired fasting glycaemia; IGT, impaired glucose tolerance; OR, odds ratio.

Model adjusted for demographic characteristics (sex, age group, race, education, marital status); BMI category, smoking status, drinking status, hypertension and diabetes.

Pelvic cancer: including cancers of the prostate, bladder, colon, cervix (cervical), uterus (uterine), rectum (rectal), and ovary (ovarian).

Non-pelvic cancer: including other kinds of cancer in addition to pelvic cancer.
