# Supplementary material for: Association of cancer with overactive bladder and impact of overactive bladder on mortality among cancer survivors: NHANES 1999-2018
Source: PLoS One. 2025 Apr 15;20(4):e0320491. doi: 10.1371/journal.pone.0320491 (PMC11999114; doi:10.1371/journal.pone.0320491)
Supplement: Table S3 — (DOCX) [file pone.0320491.s003.docx]

**Table S3**. Association of overactive bladder with all-cause death among participants without cancer.

| **Variable** | **HR (95% CI)** | ***P* value** |
| --- | --- | --- |
| Overactive bladder |  |  |
| No | ref | ref |
| Yes | 1.42 (1.25, 1.60) | < 0.0001 |
| Sex |  |  |
| Female | ref | ref |
| Male | 1.63 (1.47, 1.81) | < 0.0001 |
| Age group |  |  |
| ≤49 | ref | ref |
| 50-65 | 3.10 (2.52, 3.80) | < 0.0001 |
| ≥65 | 11.06 (8.96, 13.67) | < 0.0001 |
| Race |  |  |
| Hispanic | ref | ref |
| Non-Hispanic White | 1.84 (1.44, 2.34) | < 0.0001 |
| Non-Hispanic Black | 1.47 (1.13, 1.90) | 0.003 |
| Mexican American | 1.01 (0.77, 1.33) | 0.92 |
| Other | 1.14 (0.82, 1.57) | 0.44 |
| Education |  |  |
| Less than high school | ref | ref |
| High school or equivalent | 0.88 (0.76, 1.02) | 0.09 |
| Some college or AA degree | 0.84 (0.73, 0.96) | 0.01 |
| College graduate or above | 0.60 (0.50, 0.72) | < 0.0001 |
| Marital status |  |  |
| Divorced | ref | ref |
| Living with partner | 1.01 (0.74, 1.38) | 0.94 |
| Married | 0.73 (0.63, 0.85) | < 0.0001 |
| Never married | 1.10 (0.84, 1.43) | 0.50 |
| Separated | 1.22 (0.87, 1.71) | 0.26 |
| Widowed | 1.60 (1.34, 1.93) | < 0.0001 |
| BMI category |  |  |
| <25 | ref | ref |
| 25-30 | 0.70 (0.61, 0.81) | < 0.0001 |
| ≥30 | 0.75 (0.66, 0.86) | < 0.0001 |
| Smoking status |  |  |
| Never | ref | ref |
| Former | 1.23 (1.08, 1.40) | 0.001 |
| Now | 1.99 (1.75, 2.25) | < 0.0001 |
| Drinking status |  |  |
| Never | ref | ref |
| Former | 1.19 (0.97, 1.46) | 0.09 |
| Now | 0.69 (0.58, 0.83) | < 0.0001 |
| Hypertension |  |  |
| No | ref | ref |
| Yes | 1.58 (1.40, 1.78) | < 0.0001 |
| Diabetes |  |  |
| No | ref | ref |
| IGT | 1.19 (0.97, 1.47) | 0.10 |
| IFG | 1.32 (1.03, 1.68) | 0.03 |
| DM | 1.64 (1.43, 1.87) | < 0.0001 |

BMI, body mass index; CI, confidence interval; DM, diabetes mellitus; HR, hazard ratio; IFG, impaired fasting glycaemia; IGT, impaired glucose tolerance.

Model adjusted for demographic characteristics (sex, age group, race, education, marital status); BMI category, smoking status, drinking status, hypertension and diabetes.
