# Supplementary material for: Association of cancer with overactive bladder and impact of overactive bladder on mortality among cancer survivors: NHANES 1999-2018
Source: PLoS One. 2025 Apr 15;20(4):e0320491. doi: 10.1371/journal.pone.0320491 (PMC11999114; doi:10.1371/journal.pone.0320491)
Supplement: Table S4 — (DOCX) [file pone.0320491.s004.docx]

**Table S4.** Association of overactive bladder with all-cause mortality among participants with GU cancer.

| **Variable** | **HR (95% CI)** | ***P* value** |
| --- | --- | --- |
| Overactive bladder |  |  |
| No | ref | ref |
| Yes | 2.16 (1.47, 3.15) | < 0.0001 |
| Sex |  |  |
| Female | ref | ref |
| Male | 0.57 (0.31, 1.07) | 0.08 |
| Age group |  |  |
| ≤49 | ref | ref |
| 50-65 | 0.51 (0.10, 2.74) | 0.43 |
| ≥65 | 2.54 (0.52,12.37) | 0.25 |
| Race |  |  |
| Hispanic | ref | ref |
| Non-Hispanic White | 2.60 (1.12, 6.05) | 0.03 |
| Non-Hispanic Black | 1.02 (0.42, 2.50) | 0.97 |
| Mexican American | 1.27 (0.41, 3.93) | 0.68 |
| Other | 1.57 (0.45, 5.50) | 0.48 |
| Education |  |  |
| Less than high school | ref | ref |
| High school or equivalent | 1.09 (0.72, 1.66) | 0.69 |
| Some college or AA degree | 0.57 (0.35, 0.92) | 0.02 |
| College graduate or above | 0.80 (0.47, 1.36) | 0.40 |
| Marital status |  |  |
| Divorced | ref | ref |
| Living with partner | 0.07 (0.01, 0.64) | 0.02 |
| Married | 0.41 (0.23, 0.75) | 0.004 |
| Never married | 0.29 (0.07, 1.16) | 0.08 |
| Separated | 0.67 (0.29, 1.58) | 0.37 |
| Widowed | 0.66 (0.34, 1.30) | 0.23 |
| BMI category |  |  |
| <25 | ref | ref |
| 25-30 | 0.71 (0.46, 1.09) | 0.11 |
| ≥30 | 1.00 (0.61, 1.66) | 0.99 |
| Smoking status |  |  |
| Never | ref | ref |
| Former | 1.75 (1.07, 2.87) | 0.03 |
| Now | 4.19 (2.19, 8.01) | < 0.0001 |
| Drinking status |  |  |
| Never | ref | ref |
| Former | 0.60 (0.29, 1.27) | 0.18 |
| Now | 0.35 (0.17, 0.71) | 0.004 |
| Hypertension |  |  |
| No | ref | ref |
| Yes | 1.41 (0.98, 2.02) | 0.06 |
| Diabetes |  |  |
| No | ref | ref |
| IGT | 0.83 (0.36, 1.88) | 0.65 |
| IFG | 0.40 (0.18, 0.91) | 0.03 |
| DM | 1.12 (0.74, 1.71) | 0.59 |

BMI, body mass index; CI, confidence interval; DM, diabetes mellitus; HR, hazard ratio; IFG, impaired fasting glycaemia; IGT, impaired glucose tolerance.

Model adjusted for demographic characteristics (sex, age group, race, education, marital status); BMI category, smoking status, drinking status, hypertension and diabetes.

GU cancer: genitourinary cancer, including cancers of the kidney, prostate, bladder, and testis.
