# Supplementary material for: Association of cancer with overactive bladder and impact of overactive bladder on mortality among cancer survivors: NHANES 1999-2018
Source: PLoS One. 2025 Apr 15;20(4):e0320491. doi: 10.1371/journal.pone.0320491 (PMC11999114; doi:10.1371/journal.pone.0320491)
Supplement: Table S5 — (DOCX) [file pone.0320491.s005.docx]

**Table S5.** Association of overactive bladder with all-cause mortality among participants with Non-GU cancer.

| **Variable** | **HR (95% CI)** | ***P* value** |
| --- | --- | --- |
| Overactive bladder |  |  |
| No | ref | ref |
| Yes | 1.63 (1.34, 1.97) | < 0.0001 |
| Sex |  |  |
| Female | ref | ref |
| Male | 1.87 (1.49, 2.36) | < 0.0001 |
| Age group |  |  |
| ≤49 | ref | ref |
| 50-65 | 3.00 (1.46, 6.18) | 0.003 |
| ≥65 | 7.92 (4.06,15.44) | < 0.0001 |
| Race |  |  |
| Hispanic | ref | ref |
| Non-Hispanic White | 2.67 (1.35, 5.28) | 0.005 |
| Non-Hispanic Black | 3.22 (1.59, 6.53) | 0.001 |
| Mexican American | 1.93 (0.92, 4.05) | 0.08 |
| Other | 2.77 (1.11, 6.93) | 0.03 |
| Education |  |  |
| Less than high school | ref | ref |
| High school or equivalent | 0.77 (0.58, 1.02) | 0.07 |
| Some college or AA degree | 0.67 (0.49, 0.92) | 0.01 |
| College graduate or above | 0.52 (0.40, 0.68) | < 0.0001 |
| Marital status |  |  |
| Divorced | ref | ref |
| Living with partner | 0.63 (0.30, 1.32) | 0.22 |
| Married | 0.61 (0.46, 0.81) | < 0.001 |
| Never married | 1.02 (0.67, 1.55) | 0.93 |
| Separated | 0.69 (0.34, 1.42) | 0.32 |
| Widowed | 1.30 (0.97, 1.75) | 0.08 |
| BMI category |  |  |
| <25 | ref | ref |
| 25-30 | 0.70 (0.54, 0.91) | 0.01 |
| ≥30 | 0.56 (0.43, 0.74) | < 0.0001 |
| Smoking status |  |  |
| Never | ref | ref |
| Former | 1.29 (1.03, 1.61) | 0.02 |
| Now | 1.66 (1.24, 2.21) | < 0.001 |
| Drinking status |  |  |
| Never | ref | ref |
| Former | 1.11 (0.83, 1.47) | 0.49 |
| Now | 0.66 (0.48, 0.90) | 0.01 |
| Hypertension |  |  |
| No | ref | ref |
| Yes | 1.69 (1.32, 2.16) | < 0.0001 |
| Diabetes |  |  |
| No | ref | ref |
| IGT | 1.33 (0.87, 2.04) | 0.19 |
| IFG | 1.12 (0.65, 1.91) | 0.69 |
| DM | 1.42 (1.11, 1.81) | 0.005 |

BMI, body mass index; CI, confidence interval; DM, diabetes mellitus; HR, hazard ratio; IFG, impaired fasting glycaemia; IGT, impaired glucose tolerance.

Model adjusted for demographic characteristics (sex, age group, race, education, marital status); BMI category, smoking status, drinking status, hypertension and diabetes.

Non-GU cancer: non-genitourinary cancer, including other kinds of cancer in addition to GU cancer.
