# Supplementary material for: Association of cancer with overactive bladder and impact of overactive bladder on mortality among cancer survivors: NHANES 1999-2018
Source: PLoS One. 2025 Apr 15;20(4):e0320491. doi: 10.1371/journal.pone.0320491 (PMC11999114; doi:10.1371/journal.pone.0320491)
Supplement: Table S6 — (DOCX) [file pone.0320491.s006.docx]

**Table S6.** Association of overactive bladder with all-cause mortality among participants with pelvic cancer.

| **Variable** | **HR (95% CI)** | ***P* value** |
| --- | --- | --- |
| Overactive bladder |  |  |
| No | ref | ref |
| Yes | 1.73 (1.23, 2.42) | 0.002 |
| Sex |  |  |
| Female | ref | ref |
| Male | 1.63 (1.20, 2.22) | 0.002 |
| Age group |  |  |
| ≤49 | ref | ref |
| 50-65 | 4.07 (1.65,10.06) | 0.002 |
| ≥65 | 11.37 (4.72,27.37) | < 0.0001 |
| Race |  |  |
| Hispanic | ref | ref |
| Non-Hispanic White | 4.53 (2.06, 9.98) | < 0.001 |
| Non-Hispanic Black | 2.90 (1.24, 6.74) | 0.01 |
| Mexican American | 2.34 (0.95, 5.75) | 0.07 |
| Other | 4.00 (1.44,11.09) | 0.01 |
| Education |  |  |
| Less than high school | ref | ref |
| High school or equivalent | 0.75 (0.51, 1.10) | 0.14 |
| Some college or AA degree | 0.93 (0.67, 1.28) | 0.65 |
| College graduate or above | 0.90 (0.60, 1.33) | 0.59 |
| Marital status |  |  |
| Divorced | ref | ref |
| Living with partner | 0.29 (0.10, 0.82) | 0.02 |
| Married | 0.48 (0.30, 0.75) | 0.001 |
| Never married | 0.47 (0.21, 1.04) | 0.06 |
| Separated | 0.78 (0.39, 1.54) | 0.47 |
| Widowed | 0.95 (0.54, 1.66) | 0.85 |
| BMI category |  |  |
| <25 | ref | ref |
| 25-30 | 0.78 (0.56, 1.10) | 0.16 |
| ≥30 | 0.80 (0.56, 1.15) | 0.23 |
| Smoking status |  |  |
| Never | ref | ref |
| Former | 1.46 (1.01, 2.09) | 0.04 |
| Now | 1.96 (1.13, 3.41) | 0.02 |
| Drinking status |  |  |
| Never | ref | ref |
| Former | 1.03 (0.68, 1.55) | 0.89 |
| Now | 0.58 (0.38, 0.89) | 0.01 |
| Hypertension |  |  |
| No | ref | ref |
| Yes | 1.72 (1.27, 2.35) | < 0.001 |
| Diabetes |  |  |
| No | ref | ref |
| IGT | 0.94 (0.56, 1.57) | 0.81 |
| IFG | 0.63 (0.34, 1.17) | 0.14 |
| DM | 1.42 (1.05, 1.92) | 0.02 |

BMI, body mass index; CI, confidence interval; DM, diabetes mellitus; HR, hazard ratio; IFG, impaired fasting glycaemia; IGT, impaired glucose tolerance.

Model adjusted for demographic characteristics (sex, age group, race, education, marital status); BMI category, smoking status, drinking status, hypertension and diabetes.

Pelvic cancer: including cancers of the prostate, bladder, colon, cervix (cervical), uterus (uterine), rectum (rectal), and ovary (ovarian).
