# Supplementary material for: Association of cancer with overactive bladder and impact of overactive bladder on mortality among cancer survivors: NHANES 1999-2018
Source: PLoS One. 2025 Apr 15;20(4):e0320491. doi: 10.1371/journal.pone.0320491 (PMC11999114; doi:10.1371/journal.pone.0320491)
Supplement: Table S7 — (DOCX) [file pone.0320491.s007.docx]

**Table S7.** Association of overactive bladder with all-cause mortality among participants with non-pelvic cancer.

| **Variable** | **HR (95% CI)** | ***P* value** |
| --- | --- | --- |
| Overactive bladder |  |  |
| No | ref | ref |
| Yes | 1.68 (1.36, 2.07) | < 0.0001 |
| Sex |  |  |
| Female | ref | ref |
| Male | 1.80 (1.39, 2.32) | < 0.0001 |
| Age group |  |  |
| ≤49 | ref | ref |
| 50-65 | 2.47 (1.08, 5.65) | 0.03 |
| ≥65 | 7.30 (3.34,15.96) | < 0.0001 |
| Race |  |  |
| Hispanic | ref | ref |
| Non-Hispanic White | 1.90 (0.95, 3.80) | 0.07 |
| Non-Hispanic Black | 2.52 (1.25, 5.09) | 0.01 |
| Mexican American | 1.59 (0.71, 3.55) | 0.26 |
| Other | 1.95 (0.74, 5.15) | 0.18 |
| Education |  |  |
| Less than high school | ref | ref |
| High school or equivalent | 0.83 (0.60, 1.14) | 0.25 |
| Some college or AA degree | 0.58 (0.41, 0.81) | 0.002 |
| College graduate or above | 0.46 (0.34, 0.62) | < 0.0001 |
| Marital status |  |  |
| Divorced | ref | ref |
| Living with partner | 0.68 (0.26, 1.77) | 0.42 |
| Married | 0.65 (0.44, 0.95) | 0.03 |
| Never married | 1.14 (0.74, 1.75) | 0.56 |
| Separated | 0.74 (0.31, 1.77) | 0.50 |
| Widowed | 1.32 (0.89, 1.95) | 0.16 |
| BMI category |  |  |
| <25 | ref | ref |
| 25-30 | 0.68 (0.52, 0.90) | 0.01 |
| ≥30 | 0.56 (0.41, 0.76) | < 0.001 |
| Smoking status |  |  |
| Never | ref | ref |
| Former | 1.34 (1.07, 1.68) | 0.01 |
| Now | 1.81 (1.21, 2.69) | 0.004 |
| Drinking status |  |  |
| Never | ref | ref |
| Former | 1.00 (0.71, 1.40) | 1.00 |
| Now | 0.62 (0.44, 0.87) | 0.01 |
| Hypertension |  |  |
| No | ref | ref |
| Yes | 1.56 (1.20, 2.04) | 0.001 |
| Diabetes |  |  |
| No | ref | ref |
| IGT | 1.45 (0.94, 2.22) | 0.09 |
| IFG | 1.12 (0.58, 2.14) | 0.74 |
| DM | 1.31 (0.99, 1.74) | 0.06 |

BMI, body mass index; CI, confidence interval; DM, diabetes mellitus; HR, hazard ratio; IFG, impaired fasting glycaemia; IGT, impaired glucose tolerance.

Model adjusted for demographic characteristics (sex, age group, race, education, marital status); BMI category, smoking status, drinking status, hypertension and diabetes.

Non-pelvic cancer: including other kinds of cancer in addition to pelvic cancer.
