# Supplementary material for: Association of cancer with overactive bladder and impact of overactive bladder on mortality among cancer survivors: NHANES 1999-2018
Source: PLoS One. 2025 Apr 15;20(4):e0320491. doi: 10.1371/journal.pone.0320491 (PMC11999114; doi:10.1371/journal.pone.0320491)
Supplement: Table S8 — (DOCX) [file pone.0320491.s008.docx]

**Table S8.** Association of overactive bladder with cancer-specific mortality among participants with cancer.

| Variable | HR (95% CI) | *P* value |
| --- | --- | --- |
| Overactive bladder |  |  |
| No | ref | ref |
| Yes | 1.65 (1.29, 2.12) | < 0.0001 |
| Sex |  |  |
| Female | ref | ref |
| Male | 1.46 (1.15, 1.86) | 0.002 |
| Age group |  |  |
| ≤49 | ref | ref |
| 50-65 | 3.97 (2.38, 6.64) | < 0.0001 |
| ≥65 | 13.73 (8.86,21.29) | < 0.0001 |
| Race |  |  |
| Hispanic | ref | ref |
| Non-Hispanic White | 2.22 (1.38, 3.55) | < 0.001 |
| Non-Hispanic Black | 1.50 (0.88, 2.54) | 0.13 |
| Mexican American | 1.24 (0.74, 2.09) | 0.41 |
| Other | 1.87 (1.00, 3.49) | 0.05 |
| Education |  |  |
| Less than high school | ref | ref |
| High school or equivalent | 0.90 (0.69, 1.16) | 0.41 |
| Some college or AA degree | 0.87 (0.69, 1.09) | 0.23 |
| College graduate or above | 0.62 (0.45, 0.85) | 0.003 |
| Marital status |  |  |
| Divorced | ref | ref |
| Living with partner | 0.63 (0.29, 1.37) | 0.24 |
| Married | 0.71 (0.49, 1.02) | 0.06 |
| Never married | 1.45 (0.87, 2.42) | 0.15 |
| Separated | 0.65 (0.33, 1.27) | 0.21 |
| Widowed | 1.37 (0.91, 2.05) | 0.13 |
| BMI category |  |  |
| <25 | ref | ref |
| 25-30 | 0.76 (0.60, 0.97) | 0.03 |
| ≥30 | 0.94 (0.73, 1.22) | 0.65 |
| Smoking status |  |  |
| Never | ref | ref |
| Former | 1.53 (1.21, 1.93) | < 0.001 |
| Now | 1.99 (1.49, 2.64) | < 0.0001 |
| Drinking status |  |  |
| Never | ref | ref |
| Former | 1.01 (0.74, 1.38) | 0.93 |
| Now | 0.62 (0.47, 0.82) | < 0.001 |
| Hypertension |  |  |
| No | ref | ref |
| Yes | 1.18 (0.92, 1.50) | 0.19 |
| Diabetes |  |  |
| No | ref | ref |
| IGT | 1.25 (0.86, 1.82) | 0.24 |
| IFG | 1.88 (1.22, 2.90) | 0.004 |
| DM | 1.49 (1.15, 1.92) | 0.002 |

BMI, body mass index; CI, confidence interval; DM, diabetes mellitus; HR, hazard ratio; IFG, impaired fasting glycaemia; IGT, impaired glucose tolerance.

Model adjusted for demographic characteristics (sex, age group, race, education, marital status); BMI category, smoking status, drinking status, hypertension and diabetes.
