# Supplementary material for: Association of cancer with overactive bladder and impact of overactive bladder on mortality among cancer survivors: NHANES 1999-2018
Source: PLoS One. 2025 Apr 15;20(4):e0320491. doi: 10.1371/journal.pone.0320491 (PMC11999114; doi:10.1371/journal.pone.0320491)
Supplement: Table S9 — (DOCX) [file pone.0320491.s009.docx]

**Table S9.** Association of overactive bladder with cardiovascular disease-related mortality among participants with cancer.

| Variable | HR (95% CI) | *P* value |
| --- | --- | --- |
| Overactive bladder |  |  |
| No | ref | ref |
| Yes | 1.48 (1.32, 1.65) | < 0.0001 |
| Sex |  |  |
| Female | ref | ref |
| Male | 1.70 (1.53, 1.89) | < 0.0001 |
| Age group |  |  |
| ≤49 | ref | ref |
| 50-65 | 3.01 (2.47, 3.67) | < 0.0001 |
| ≥65 | 10.86 (8.76,13.46) | < 0.0001 |
| Race |  |  |
| Hispanic | ref | ref |
| Non-Hispanic White | 1.94 (1.50, 2.50) | < 0.0001 |
| Non-Hispanic Black | 1.56 (1.20, 2.01) | < 0.001 |
| Mexican American | 1.01 (0.75, 1.34) | 0.97 |
| Other | 1.12 (0.80, 1.58) | 0.51 |
| Education |  |  |
| Less than high school | ref | ref |
| High school or equivalent | 0.86 (0.74, 0.99) | 0.03 |
| Some college or AA degree | 0.79 (0.68, 0.93) | 0.004 |
| College graduate or above | 0.61 (0.51, 0.73) | < 0.0001 |
| Marital status |  |  |
| Divorced | ref | ref |
| Living with partner | 0.96 (0.71, 1.28) | 0.76 |
| Married | 0.71 (0.61, 0.82) | < 0.0001 |
| Never married | 0.94 (0.74, 1.19) | 0.59 |
| Separated | 1.25 (0.88, 1.77) | 0.20 |
| Widowed | 1.56 (1.32, 1.84) | < 0.0001 |
| BMI category |  |  |
| <25 | ref | ref |
| 25-30 | 0.68 (0.60, 0.78) | < 0.0001 |
| ≥30 | 0.66 (0.58, 0.74) | < 0.0001 |
| Smoking status |  |  |
| Never | ref | ref |
| Former | 1.23 (1.09, 1.38) | < 0.001 |
| Now | 1.93 (1.70, 2.20) | < 0.0001 |
| Drinking status |  |  |
| Never | ref | ref |
| Former | 1.20 (0.99, 1.46) | 0.07 |
| Now | 0.69 (0.58, 0.83) | < 0.0001 |
| Hypertension |  |  |
| No | ref | ref |
| Yes | 1.73 (1.53, 1.96) | < 0.0001 |
| Diabetes |  |  |
| No | ref | ref |
| IGT | 1.18 (0.98, 1.41) | 0.08 |
| IFG | 1.07 (0.85, 1.34) | 0.58 |
| DM | 1.58 (1.38, 1.80) | < 0.0001 |

BMI, body mass index; CI, confidence interval; DM, diabetes mellitus; HR, hazard ratio; IFG, impaired fasting glycaemia; IGT, impaired glucose tolerance.

Model adjusted for demographic characteristics (sex, age group, race, education, marital status); BMI category, smoking status, drinking status, hypertension and diabetes.
