# Supplementary material for: Association of cancer with overactive bladder and impact of overactive bladder on mortality among cancer survivors: NHANES 1999-2018
Source: PLoS One. 2025 Apr 15;20(4):e0320491. doi: 10.1371/journal.pone.0320491 (PMC11999114; doi:10.1371/journal.pone.0320491)
Supplement: Table S10 — (DOCX) [file pone.0320491.s010.docx]

**Table S10.** Association of overactive bladder with cerebrovascular disease-related mortality among participants with cancer.

| **Variable** | **HR (95% CI)** | ***P* value** |
| --- | --- | --- |
| Overactive bladder |  |  |
| No | ref | ref |
| Yes | 1.47 ( 1.32, 1.64) | < 0.0001 |
| Sex |  |  |
| Female | ref | ref |
| Male | 1.67 ( 1.49, 1.86) | < 0.0001 |
| Age group |  |  |
| ≤49 | ref | ref |
| 50-65 | 3.26 ( 2.63, 4.05) | < 0.0001 |
| ≥65 | 12.48 (10.08,15.45) | < 0.0001 |
| Race |  |  |
| Hispanic | ref | ref |
| Non-Hispanic White | 1.99 ( 1.52, 2.61) | < 0.0001 |
| Non-Hispanic Black | 1.51 ( 1.14, 2.00) | 0.004 |
| Mexican American | 1.07 ( 0.79, 1.44) | 0.67 |
| Other | 1.22 ( 0.84, 1.77) | 0.29 |
| Education |  |  |
| Less than high school | ref | ref |
| High school or equivalent | 0.85 ( 0.74, 0.99) | 0.04 |
| Some college or AA degree | 0.79 ( 0.67, 0.94) | 0.01 |
| College graduate or above | 0.62 ( 0.52, 0.74) | < 0.0001 |
| Marital status |  |  |
| Divorced | ref | ref |
| Living with partner | 0.99 ( 0.72, 1.35) | 0.94 |
| Married | 0.78 ( 0.66, 0.92) | 0.004 |
| Never married | 1.06 ( 0.81, 1.38) | 0.68 |
| Separated | 1.25 ( 0.88, 1.77) | 0.20 |
| Widowed | 1.60 ( 1.32, 1.92) | < 0.0001 |
| BMI category |  |  |
| <25 | ref | ref |
| 25-30 | 0.72 ( 0.61, 0.85) | < 0.0001 |
| ≥30 | 0.76 ( 0.66, 0.87) | < 0.0001 |
| Smoking status |  |  |
| Never | ref | ref |
| Former | 1.32 ( 1.18, 1.49) | < 0.0001 |
| Now | 2.17 ( 1.91, 2.47) | < 0.0001 |
| Drinking status |  |  |
| Never | ref | ref |
| Former | 1.19 ( 0.99, 1.42) | 0.06 |
| Now | 0.67 ( 0.57, 0.80) | < 0.0001 |
| Hypertension |  |  |
| No | ref | ref |
| Yes | 1.57 ( 1.39, 1.78) | < 0.0001 |
| Diabetes |  |  |
| No | ref | ref |
| IGT | 1.04 ( 0.86, 1.25) | 0.72 |
| IFG | 1.24 ( 0.99, 1.55) | 0.06 |
| DM | 1.52 ( 1.34, 1.72) | < 0.0001 |

BMI, body mass index; CI, confidence interval; DM, diabetes mellitus; HR, hazard ratio; IFG, impaired fasting glycaemia; IGT, impaired glucose tolerance.

Model adjusted for demographic characteristics (sex, age group, race, education, marital status); BMI category, smoking status, drinking status, hypertension and diabetes.
