# Supplementary material for: Association of cancer with overactive bladder and impact of overactive bladder on mortality among cancer survivors: NHANES 1999-2018
Source: PLoS One. 2025 Apr 15;20(4):e0320491. doi: 10.1371/journal.pone.0320491 (PMC11999114; doi:10.1371/journal.pone.0320491)
Supplement: Table S11 — (DOCX) [file pone.0320491.s011.docx]

| Cause of death | Total (n = 3,090) | Individuals without overactive bladder (n = 2,046) | Individuals with overactive bladder (n = 1,044) |
| --- | --- | --- | --- |
|  |  |  |  |
| Malignant neoplasms | 289 | 149 | 140 |
| Cardiovascular disease | 173 | 98 | 75 |
| Cerebrovascular disease | 41 | 26 | 15 |
| Accidents | 25 | 15 | 10 |
| Alzheimer's disease | 24 | 15 | 9 |
| Influenza and pneumonia | 17 | 9 | 8 |
| Diabetes mellitus | 14 | 4 | 10 |
| All other causes | 267 | 137 | 130 |

**Table S11.** Causes of death among participants with cancer.
