# Supplementary material for: Association of cancer with overactive bladder and impact of overactive bladder on mortality among cancer survivors: NHANES 1999-2018
Source: PLoS One. 2025 Apr 15;20(4):e0320491. doi: 10.1371/journal.pone.0320491 (PMC11999114; doi:10.1371/journal.pone.0320491)
Supplement: Attachment — Submitted filename: Response to reviewers.docx [file pone.0320491.s013.docx]

**Reviewer #1:**

**Comment 1:** I congratulate the authors for the study conducted on a large sample. However, I have some suggestions regarding the article.

**Answer 1:** We greatly appreciate the reviewer’s valuable comment. We would like to revise the manuscript carefully according to the reviewer’s insightful suggestion.

**Comment 2:** The relationship between mortality due to cerebrovascular and cardiovascular diseases is given in Tables S8-10, but data on patient numbers can be added to Table 1.

**Answer 2:** Thank you very much for the comment. We provide the association of overactive bladder with cancer-specific mortality, cardiovascular disease-related mortality and cerebrovascular disease-related mortality among participants with cancer in Table S8-10. In Table 1, we provide the general characteristics of individuals stratified by the presence or absence of overactive bladder. We greatly appreciate the reviewer’s valuable suggestion, but we think it is not proper to add these patient numbers in Table 1. Instead, we add another table (Table S11) to show the exact patient numbers about the causes of death among participants with cancer.

**Comment 3****:** The study analyzed the relationship between mortality due to cerebrovascular and cardiovascular diseases. Were there any patients with diseases that could affect mortality, such as chronic obstructive pulmonary disease, chronic liver disease, and chronic kidney disease, in the patient group included in the study? If so, why were they not included in the analysis?

**Answer 3:** Thank you very much for the insightful comment. Actually, these participants might have multiple diseases that could affect mortality. However, it is impossible to have detailed information of each participant due to the intrinsic limitations of NHANES database. Thus, we stated in the Discussion section that there may still be residual confounding from unmeasured or unknown factors. Despite these constraints, our investigation unveiled a positive association between cancer, OAB and mortality. We greatly appreciate the reviewer’s comment and have added these potential factors in the Discussion section.

**Comment 4:** What were the treatments for overactive bladder? Were there any patients who did not receive OAB treatment?

**Answer 4:** Thank you very much for the considerate comment. However, the NHANES database does not provide detailed information about the treatments for overactive bladder.

**Comment 5:** The relationship between mortality and side effects of the chemotherapeutic agents used can be analyzed (e.g. kidney damage due to cisplatin use).

**Answer 5:** We greatly appreciate the reviewer’s insightful comment. The relationship between mortality and side effects of the chemotherapeutic agents is a meaningful topic and warrants further investigation. However, the NHANES database did not provide such information. Thus, we could not explore such relationship using the NHANES database. We will pay attention to this question and try to investigate the relationship in future studies.

**Comment 6:** In the discussion section, additional information about the lower urinary tract symptoms that may occur due to radiotherapy and some chemotherapeutics in cancer patients can be given.

**Answer 6:** Thank you very much for the valuable suggestion. We have added the statement about the lower urinary tract symptoms due to radiotherapy and some chemotherapeutics in the Discussion section.

**Reviewer #2: The authors determine the correlation between cancer disease and a chronic syndrome of over active bladder. They question "the impact of OAB on mortality". I appreciate that the authors try to increase the awareness for OAB in this patient group. I have some questions and comments.**

**Introduction:**
**Comment 1:** "there is a pressing need ... to improve their survival...". Is it really only to improve survival or to get awareness of OAB in these patients to adequately treat these bothersome complaints and increase quality of life in cancer disease/survivors?

**Answer 1:** Thank you very much for the constructive comment. We have revised the Introduction section according to the reviewer’s valuable suggestion.

**Comment 2:** Is the hypothesis of this study that OAB (as risk factors) increase mortality?
**Answer 2:** We totally agree with the reviewer. The initial hypothesis is that OAB might increase mortality in patients with cancer. After accounting for multiple covariates, OAB was associated with an elevated risk of all-cause and cancer-specific mortality among participants diagnosed with cancer.

**Methods:**
**Comment 3:** NHANES cycles spanning from 1999-2000 to 2017-2018 => mention exact start/closure.

**Answer 3:** We greatly appreciate the reviewer’s considerate comment. However, the most recent NHANES began in 1999, and there are two years in one cycle (https://wwwn.cdc.gov/nchs/nhanes/default.aspx). The NHANES database did not mention exact start/closure. Instead, we mention the NHANES cycles which were used in our study.

**Comment 4:** Remove "variable meq220), especially as the database have not been made available in the submission or been mention in what way available correctly.

**Answer 4:** We completely agree with the reviewer and have removed “variable mcq220” in the sentence.

**Comment 5:** Outcome variable: validated questions on its own?

**Answer 5:** Thank you for the insightful comment. The outcome variable in the present study is OAB. However, the evaluation of OAB involved these specific questions. Actually, these questions were obtained from the NHANES (https://wwwn.cdc.gov/Nchs/Nhanes/2009-2010/KIQ_U_F.htm#KIQ042). We provided the questions in our manuscript. We cherish the hope that other researchers would understand the definition of OAB better.

**Comment 6:** OABSS, "a rigorously"=> validated? Cumulative OABSS of UUI and nocturia score=> validated outcome?

**Answer 6:** Thank you very much for the comment. Actually, OABSS is a rigorously developed questionnaire and has been validated by many previous studies. We are sorry for the confusion and have revised this sentence.

**Comment 7:** Covariate assessment/statistical analysis: 4x "meticulously". Avoid subjective words in methods/results anyway.
**Answer 7:** We totally agree with the reviewer and have deleted these subjective words in the revised manuscript.

**Results:**
**Comment 8: "9.606 and 20.167%=> 10 and 20% or 9.6 and 20.2%. Idem for mean age/BMI etcetera.**

**Answer 8:** Thank you very much for the constructive comment. We have revised the manuscript according to the reviewer’s valuable suggestion.

**Comment 9:** Individuals with OAB had more know risk factors for OAB: age, BMI, diabetes mellitus, alcohol consumption, hypertension. => implies that the cancer disease population is a more vulnerable population with more morbidity and risk factors for OAB? And OAB symptoms to be aware of as it is already suspected to occur more in a patient?

A**nswer 9: W**e totally agree with the reviewer that the cancer disease population is a more vulnerable population with more morbidity and risk factors for OAB. By analyzing the risk factors of OAB, we could identify patients with OAB earlier.

**Comment 10:** Relationship between cancer status and OAB: Across model: not explanted in text methods and not in al tables.

**Answer 10:** Thank you very much for the comment. We have explained the relationship between cancer status and OAB in the “Statistical Analysis” of Methods section. Moreover, the relationship across models was also shown in Table 2 and Results section.

**Comment 11:** "we categorized cancer patients..." and "Furthermore, the study participants..."=> methods section.
**Answer 11:** We greatly appreciate the valuable comment and have moved these two sentences to the Methods section.

**Discussion:**
**Comment 12:** "demonstrated a robust association between OAB presence and heightened mortality": OAB not an entity/disease itself. No causality. Correlation the other way around? Cancer patients more vulnerable and having more risk factors to get OAB complaints?
**Answer 12:** Thank you very much for the constructive comment. We totally agree with the reviewer and have revised this paragraph to make it make more plausible.

**Tables/figures:**
**Comment 13:** Figure 3: units at axes

**Answer13:** We greatly appreciate the reviewer’s constructive comment and have added units at axes.
