# Supplementary material for: Association of cancer with overactive bladder and impact of overactive bladder on mortality among cancer survivors: NHANES 1999-2018
Source: PLoS One. 2025 Apr 15;20(4):e0320491. doi: 10.1371/journal.pone.0320491 (PMC11999114; doi:10.1371/journal.pone.0320491)
Supplement: Attachment — Submitted filename: Response_to_Reviewers_auresp_2.docx [file pone.0320491.s014.docx]

**Reviewer #3:**

**Comment 1**. In the NHANES database, we often take PIR as an important covariate, which is recommended to be included;

**Answer 1:** Thank you very much for the insightful comment. We appreciate your reference to the Family Income-to-Poverty Ratio (PIR) as an index of socioeconomic standing, which has indeed been utilized in some NHANES studies. However, in the context of our current research, our primary focus is on elucidating the associations between cancer and the risk of Overactive Bladder (OAB), as well as the links between OAB and mortality outcomes among cancer survivors. Drawing on our extensive clinical experience, we believe that PIR may not significantly influence these specific relationships. Instead, we have incorporated a comprehensive set of other important and relevant variables, such as gender, race, education, marital status, BMI, smoking status, drinking status, and history of hypertension and diabetes. We are confident that these combined variables collectively provide a more nuanced and accurate representation of an individual's socioeconomic standing in the context of our study.uss

**Comment** 2. BMI as an important risk factor, the team has considered the impact of BMI as a categorical variable on the outcome, but is the impact of BMI as a continuous variable still highly consistent?

**Answer 2:** Thank you very much for your valuable feedback. In response to your comment, we have conducted a reanalysis of our data to thoroughly assess the impact of BMI as a continuous variable. Our findings indicate that the influence of BMI remains highly consistent when treated as a continuous variable. We recognize that categorizing BMI into distinct groups aligns well with established clinical guidelines and enhances the interpretability of results for both researchers and practitioners. Given these important considerations, we have chosen to maintain BMI as a categorical variable in the present study.

**Comment** 3. does OAB value obey normal distribution and what test method is used?

**Answer 3:** We sincerely appreciate the reviewer’s insightful comment. To assess the distribution of OAB values, we employed the Kolmogorov-Smirnov test and found that the overall OAB values do not follow a normal distribution. In our study, OAB is defined such that participants with a cumulative OAB Symptom Score (OABSS) ≥ 3 are categorically diagnosed with OAB. Among the 32,166 participants, 25,679 did not have OAB, while 6,487 were diagnosed with OAB. Notably, among those with OAB, the majority had an OAB score of 3, with fewer patients having scores of 4 or higher. Although the OAB values do not conform to a normal distribution, we believe this pattern is highly representative of the clinical reality. It aligns well with our extensive clinical experience, reflecting the typical distribution of OAB severity that we commonly encounter in practice.

**Comment** 4. is there any missing value in the data included in this article? If yes, what method is used to deal with missing values and what is the basis?

**Answer 4:** Thank you very much for your valuable comment. We fully agree with the reviewer that the initial dataset contains missing values due to the inherent limitations of the NHANES database, which could potentially influence subsequent analyses. In preparation for this study, we reviewed numerous articles focusing on NHANES data and observed how other researchers addressed similar challenges. To ensure the robustness and reliability of our findings, we made a decision to include only adults who had complete information on cancer status, OAB, socioeconomic, and health-related characteristics. We recognize that this approach may introduce some selection bias, and we have carefully addressed these limitations in detail in the Discussion section of our manuscript.

**Comment** 5. the relationship between risk factors and outcomes is significantly positive, but is this relationship linear or nonlinear?

**Answer 5:** We are very grateful for the reviewer’s considerate comment. We concur with the reviewer that the relationship between risk factors and outcomes is significantly positive. However, based on our study design and the results we have obtained, we have determined that this relationship is generally nonlinear.

**Comment** 6. the blood pressure value of hypertensive people is: systolic blood pressure greater than or equal to 140mmHg and diastolic blood pressure greater than 90mmHg. Is this standard appropriate?

**Answer 6:** Thank you very much for your valuable comment. The criteria for defining blood pressure values in hypertensive individuals are consistent with those used in numerous previous studies, treatment guidelines, and clinical practice.

**Comment** 7. is there any basis for selecting 50 and 65 years old as nodes in Table 2?

**Answer 7:** Thank you very much for your considerate comment. We fully agree with the reviewer that any choice of age nodes may introduce some bias. However, our decision to select 50 and 65 years as the nodes is based on extensive review of relevant studies. We believe that this categorization aligns more closely with clinical practice and provides a meaningful framework for our analysis.

**Comment** 8. in Figure 2, the interaction test of gender, age, hypertension and diabetes is less than 0.05. Can existing theories or potential mechanisms explain this result

**Answer 8:** We greatly appreciate the reviewer’s considerate comment. We fully agree with the reviewer that the interaction tests for gender, age, hypertension, and diabetes all yielded p-values less than 0.05. These findings suggest significant interactions among these variables, which we believe provide important insights and directions for future research. We emphasize that further epidemiological and mechanistic studies are needed to more comprehensively explain these results and elucidate the underlying mechanisms.

**Reviewer #4:** This paper aimed to explore the association of cancer with overactive bladder and impact of overactive bladder on mortality among cancer survivors by using tne data from NHANES 1999-2018. This work is interesting, but some issues came to my attention.
**Comment** 1. Complex Survey Design: As highlighted on the CDC/NHANES website, "For NHANES datasets, the use of sampling weights and sample design variables is necessary to obtain unbiased estimates and accurate standard errors and confidence intervals." The authors must use the complex survey design parameters—specifically strata, clusters, and weights—to ensure valid and reliable results.

**Answer 1:** Thank you very much for the constructive comment. We totally agreed with the reviewer and have employed suitable weighting procedures to derive nationally representative estimates.

**Comment** 2. Missing Data Analysis: The authors must address the issue of missing data by conducting a thorough missing data analysis. Tables should be included to display the percentage of missing values and the patterns of missingness, especially given that the percentage of missing data may likely exceed 40%.

**Answer 2:** We greatly appreciate the reviewer’s considerate comment. We fully agree that the initial dataset contains missing values due to the inherent limitations of the NHANES database, and addressing this issue is crucial for evaluating the robustness of our results. To provide transparency, we have clearly illustrated the selection of participants with complete data in Figure 1. Additionally, we have addressed the issue of missing data and provided the percentage of missing values in the revised manuscript. Moreover, we have thoroughly discussed these limitations and their potential impact in the Discussion section of our manuscript.

**Comment** 3. There are numerous indicators in the NHANES database, which can be reached a relationship between each other. How did you accout for the coincidence possibility of your work?

**Answer 3:** Thank you very much for the constructive comment. We completely agree with the reviewer that the NHANES database contains a vast array of indicators, many of which may be interrelated. In our clinical practice, we have encountered numerous cancer patients who also exhibit symptoms of OAB. These symptoms may arise from the cancer itself or from cancer-related treatments, including various medications. However, the precise relationship between cancer, OAB, and their impact on patient survival remains unclear. Fortunately, the NHANES database offers a valuable opportunity to explore these questions and seek answers. We believe our study will provide important insights and guidance for the treatment of cancer patients and the improvement of their prognosis.

**Comment** 4. NHANES database is a retrospective pooled tool, which is mainly obtained from the outcomes of questionnaire survey. Thus, the data may be not reliable enough to be analyzed. Some limitations regarding this issue must be specified.

**Answer 4:**

Thank you very much for your constructive comment. We fully agree with the reviewer that the NHANES database is a retrospective, pooled tool primarily derived from questionnaire survey outcomes. While it does have some limitations in terms of data analysis, it remains an invaluable resource for addressing important clinical and public health questions. Many researchers have utilized the NHANES database to explore various relationships and provide guidance for further studies. Notably, since 2010, more than 56,000 relevant studies based on NHANES data have been published. This underscores its significance in the research community.

PubMed Search for NHANES Studies (2010-2025):

<https://pubmed.ncbi.nlm.nih.gov/?term=National+Health+and+Nutrition+Examination+Survey&filter=years.2010-2025&sort=date>

**Comment** 5. Please consider to add a table to summarize the ongoing or completed clinical trials regarding this topic, which may further rich the overall contents of this paper.

**Answer 5:** We are extremely grateful for the reviewer’s thoughtful comments. We have conducted an extensive search online but were unable to locate any clinical trials that specifically focus on this topic. We believe this is an emerging area that has not yet garnered widespread attention. We are convinced that our research will provide valuable insights and meaningful directions for future clinical trials in this domain.

**Comment** 6. Please consider to include more cycles of cancer patients in the NHANES database, since current 1999-2018 cycle seemed a little outdated.

**Answer 6:** Thank you very much for the valuable suggestion. We appreciate your attention to the timeliness of the data. While it is true that the 1999-2018 cycle of the NHANES database may appear to be somewhat dated, it is essential to recognize that this period of data remains highly relevant and has been extensively utilized in numerous studies. Moreover, our research goes beyond merely examining the relationship between variables; we aim to delve into the impact of OAB on the survival of cancer patients. Including the most recent cycles of NHANES data would result in a follow-up period that is too short to accurately assess the long-term influence of OAB on the prognosis of cancer patients. Therefore, we believe that utilizing the 1999-2018 cycle is more appropriate, as it ensures the accuracy and representativeness of the cohort. Through meticulous research design and robust analytical methods, we have successfully extracted valuable insights from the existing data, providing a solid foundation for future cancer research.
